# Pin1 Is Regulated by CaMKII Activation in Glutamate-Induced Retinal Neuronal Regulated Necrosis

**DOI:** 10.3389/fncel.2019.00276

**Published:** 2019-06-25

**Authors:** Shuchao Wang, Lvshuang Liao, Yanxia Huang, Mi Wang, Hongkang Zhou, Dan Chen, Fengxia Liu, Dan Ji, Xiaobo Xia, Bing Jiang, Jufang Huang, Kun Xiong

**Affiliations:** ^1^Department of Anatomy and Neurobiology, School of Basic Medical Sciences, Central South University, Changsha, China; ^2^Raymond G. Perelman Center for Cellular and Molecular Therapeutics, The Children’s Hospital of Philadelphia, Philadelphia, PA, United States; ^3^Department of Human Anatomy, School of Basic Medical Science, Xinjiang Medical University, Ürümqi, China; ^4^Department of Ophthalmology, Xiangya Hospital, Central South University, Changsha, China; ^5^Department of Ophthalmology, The Second Xiangya Hospital, Central South University, Changsha, China

**Keywords:** regulated necrosis, glutamate, calcium, CaMKII, Pin1

## Abstract

In our previous study, we reported that peptidyl-prolyl isomerase 1 (Pin1)-modulated regulated necrosis (RN) occurred in cultured retinal neurons after glutamate injury. In the current study, we investigated the role of calcium/calmodulin-dependent protein kinase II (CaMKII) in Pin1-modulated RN in cultured rat retinal neurons, and in an animal *in vivo* model. We first demonstrated that glutamate might lead to calcium overloading mainly through ionotropic glutamate receptors activation. Furthermore, CaMKII activation induced by overloaded calcium leads to Pin1 activation and subsequent RN. Inactivation of CaMKII by KN-93 (KN, i.e., a specific CaMKII inhibitor) application can decrease the glutamate-induced retinal neuronal RN. Finally, by using an animal *in vivo* model, we also demonstrated the important role of CaMKII in glutamate-induced RN in rat retina. In addition, flash electroretinogram results provided evidence that the impaired visual function induced by glutamate can recover after CaMKII inhibition. In conclusion, CaMKII is an up-regulator of Pin1 and responsible for the RN induced by glutamate. This study provides further understanding of the regulatory pathway of RN and is a complementary mechanism for Pin1 activation mediated necrosis. This finding will provide a potential target to protect neurons from necrosis in neurodegenerative diseases, such as glaucoma, diabetic retinopathy, and even central nervous system diseases.

## Introduction

Glutamate is the most prevalent excitatory neurotransmitter in the central nervous system (CNS; Chi-Castaneda et al., 2015; Lopez-Colome et al., 2016; Madji Hounoum et al., 2018; Wang et al., 2018b). Glutamate plays a vital role in excitatory actions by gating glutamate receptors (GluRs), which are divided into ionotropic glutamate receptors (iGluRs) [*N*-methyl-D-aspartic acid receptor (NMDA), α-amino-3-hydroxy-5-methyl-4-isoxazole propionate receptor (AMPAR)/kainic acid receptor (KAR)], and metabotropic glutamate receptors (mGluRs) [mGluR1-8] (Boye Larsen et al., 2014; Guillem et al., 2015; Flores-Mendez et al., 2016; Xu et al., 2017; Liu et al., 2018). Glutamate concentration is under the control of a series of signaling cascades triggered by glutamate receptors present in the pre/post-synaptic membrane and neighboring glial cells (Lopez-Colome et al., 2016). After stimulation, glutamate is released from pre-synaptic terminals and removed by excitatory amino acid transporters and sodium-dependent glutamate transporters (Mendez-Flores et al., 2016; Chi-Castaneda et al., 2017). Meanwhile, inefficient clearance of glutamate from the synaptic cleft leads to the overstimulation of neuronal GluRs, triggering the overloaded calcium influx that activates intracellular events that result in neuronal death, such as necrosis, apoptosis, and RN, which is also known as excitotoxicity (Agudo-Barriuso et al., 2016; Chi-Castaneda et al., 2017; Madji Hounoum et al., 2017; Suarez-Pozos et al., 2017; Chi-Castaneda and Ortega, 2018).

Glutamate excitatory action has been involved in various neurodegenerative diseases including Alzheimer’s disease (AD), Parkinson’s disease (PD), as well as many retinal diseases, such as diabetic retinopathy and glaucoma (Alarcon-Martinez et al., 2010; Esposito et al., 2013; Haris et al., 2013; Singh et al., 2017; Kabra et al., 2018). Neuronal responses to glutamate treatment are characterized by increased intracellular calcium concentration through GluRs activation (Madji Hounoum et al., 2017). We have investigated the key molecular pathway involved in glutamate-induced RN in our previous study (Wang et al., 2018a, 2019). However, the specific role of distinct GluRs involved in the glutamate-induced RN in the retinal neuronal cultures has not been clarified. It has been reported that retinal neurons express all of the glutamate receptor subtypes (Luo et al., 2001; Fang et al., 2010). Some reports have indicated that retinal excitotoxicity was mediated by both non-NMDAR and NMDAR, whereas other studies have found an entirely non-NMDAR-mediated retinal neuronal death (Luo et al., 2001; Opere et al., 2018). Such differences may be partially due to the maturational changes of retinal neurons and the difference in cell culture environment (Luo et al., 2001). In the current study, we first wanted to investigate and identify the battery of glutamatergic receptors responsible for glutamate-induced RN in our retinal neuronal culture conditions.

RN is a type of programmed cell death, mainly including necroptosis, ferroptosis, parthanatos, etc. (Galluzzi et al., 2014; Chen et al., 2016; Liao et al., 2017; Xiong et al., 2017; Wang et al., 2018c, 2018d). The morphological features of RN are similar to necrosis, which exhibit plasma integrity loss and organelle swelling, and are different from apoptosis and autophagy (Galluzzi et al., 2014; Catalani et al., 2016). Both our studies and others have found that peptidyl-prolyl isomerase 1 (Pin1) activation was involved in glutamate-induced RN (Del Rosario et al., 2015; Wang et al., 2018a). Pin1 is one subtype of the peptidyl *cis-*to-*trans* isomerases (PPIases) that can bind and catalyze *cis/trans* isomerization of phosphorylated threonine/serine-proline, leading to protein phosphorylation and degradation, neuronal survival and death (Agostoni et al., 2016; Islam et al., 2018). Further reports have indicated that PPIases, including Pin1, are involved in calcium-mediated pathological and physiological changes (Ghosh et al., 2013; Agostoni et al., 2016). However, the mechanism of Pin1 activation induced by increased calcium has not been fully elucidated. It is well known that calmodulin (CaM) is a calcium-binding protein involved in many cellular physiological processes (Chai et al., 2018). CaM consists of two globular lobes that are able to bind two calcium ions, respectively (Wyttenbach et al., 2010). Calcium/calmodulin-dependent protein kinase II (CaMKII) is a protein kinase that can be regulated by the calcium/calmodulin complex (Tanaka et al., 2018). CaMKII activity could be dysregulated by increased calcium in some neuronal diseases, such as stroke, epilepsy, and glaucoma, etc. (Cooper et al., 2008; Brewster et al., 2016; Zhou et al., 2018). CaMKII inhibition prior to excitotoxic injury could prevent neuronal damage both *in vitro* and *in vivo* (Qian et al., 2019). More importantly, it has been reported that Pin1 binds with CaMKII in mouse brain homogenate (Tatara et al., 2010; Oliveira et al., 2019). However, to date it has not been reported whether Pin1 is involved in the CaMKII regulatory pathway.

Therefore, in this study we aim to investigate whether CaMKII is an up-regulator of Pin1 and whether it is responsible for the RN in cultured retinal neurons. To resolve this, the first step is to address which types of glutamate receptors are involved in the calcium changes. Then, the regulatory role of CaMKII in Pin1 mediated retinal neuronal RN will be determined. We expect that the results could provide a better understanding and rational interventional targets for neuronal RN in the future.

## Materials and Methods

### Primary Retinal Neuron Cultures and *in vitro* Model Preparation

All experimental procedures used in the present study were approved by the Ethics Committee of the 3rd Xiangya Hospital of Central South University in keeping with the Guidelines for the Care and Use of Laboratory Animals (U.S. National Institutes of Health). Primary cultures of retinal neurons were prepared from 1-day-old Sprague-Dawley (SD) rat pups as described previously (Li N. et al., 2016; Wang et al., 2017, 2018a). In brief, the eyes were removed under sterile conditions, and the retinae were extracted with the aid of a dissecting microscope. The retinae were digested at 37°C for 10 min in Dulbecco’s modified Eagle’s medium (DMEM, GE Healthcare, Logan Utah, United States) containing 0.02% papain and then the tissue was gently triturated for 20 times and filtered through a 70-mm nylon cell sieve, followed with 5-min centrifugation. After resuspension, the cells were counted and plated onto flasks or plates precoated with poly-*D*-lysine (10 μg/mL, Sigma-Aldrich, St. Louis, MO, United States) at a density of 6 × 10^5^ cells/mL. Cells were maintained in 5% CO_2_ incubator at 37°C for four hours after plating, followed by replacing the plating medium with neurobasal medium (Thermo Scientific, Waltham, MA, United States) supplemented with B27 (Thermo Scientific). Half of the culture media were replaced every 2 days. On the 7th day, the cultures were conducted with 50 μM glutamate (Sinopharm, Beijing, China) insult for indicate time points.

### Animal Model *in vivo* of Glutamate Treatment

Rats were anesthetized by intraperitoneal injection of 1% pentobarbital sodium (6 mL/kg). To dilate the pupils, chloramphenicol eye drops were placed onto the left conjunctiva sac. Intravitreal injection of a single dose of 5 μL of 100 mM glutamate in 0.01 M PBS was then conducted and a sham operation was performed as a control. Animals were allowed to survive for 12 h. Each group was composed of four animals.

### Animal Tissue Preparation

Animals were anesthetized with 1% pentobarbital sodium and perfused transcardially with 0.9% sodium chloride, followed by 4% paraformaldehyde (PF) in 0.1 M PB. Subsequently, the eyes were enucleated, anterior segments were removed, and posterior eyecups were post-fixed in 4% PF overnight at 4°C. Then, the eye cups were dehydrated in 15 and 30% sucrose in 0.1 M PB at 4°C sequentially. Next, the eyecups were embedded with Tissue-Tek optimal cutting temperature medium and frozen in liquid nitrogen. Then, 8 μm thick of cryosections were cut using a microtome (Thermo Scientific). The sections, which included the optic nerve were stored at −20°C until use.

### Drug Application

(+)-MK 801 [MK, i.e., a NMDAR antagonist (Kubrusly et al., 1998; Aihara et al., 2014), MCE, Monmouth Junction, United States], CNQX [CN, i.e., an AMPAR/KAR antagonist (Couratier et al., 1993; Thellung et al., 2013), MCE], (RS)-MCPG [MC, i.e., a non-selective group I/group II mGluRs antagonist (Kusama-Eguchi et al., 2004), MCE], BAPTA-AM (BA, i.e., a calcium chelator, Selleck, Houston, TX, United States), KN-93 Phosphate (Selleck) was dissolved in dimethyl sulfoxide (DMSO, Sigma-Aldrich) as a stock solution. The stock solution was further diluted in the medium to achieve concentration of DMSO lower than 0.1%. MK, CN, MC, BA, and KN were used at a concentration of 10, 10, 50, 10, and 10 μM, respectively. Thirty minutes before the glutamate insult, the drug solution was directly added to primary retinal neuronal cultures or injected into the vitreous cavity of rats.

### Calcium Measurement

#### Fluo-4, AM Kit

The cells were washed three times in Calcium free HBSS (Solarbio, Beijing, China) and incubated with neuronal culture media containing 4 μM Fluo-4, AM, a fluorescent probe (Solarbio) at 37°C for 20 min. The cells were then washed three times in HBSS before imaging. Images acquired with a fluorescence microscope (Olympus, Tokyo, Japan) using the same exposure time were captured from 5 random fields of each group. The excitation wavelength was 494 nm and the emission wavelength was 516 nm. Fluorescence signal intensity values of different images were equal to the calibrated integrated density, which was quantified using Image J software (NIH, Baltimore, MD, United States) divided by the number of cells.

#### Cell Calcium Assay Kit

Calcium concentrations were measured using a biochemical testing kit for cell calcium assay (Genmed, Shanghai, China) according to the manufacturer’s instructions. In brief, cells were harvested and washed three times with washing reagent. After protein lysis, the supernatants were collected and a BCA assay was used to determine the protein concentration of different groups and then diluted by using lysis reagent to form the same protein concentration samples. Serial dilutions of standard reagent (0–0.5 mM) were used to create a standard curve. For analyses, 160 μL of working reagent was added to aliquots of 20 μL of sample or standard reagent in a 96-well plate and then the plate was incubated at room temperature (RT) in the dark for 5 min, and a microplate reader (Bio-Rad, Berkeley, CA, United States) was used to measure the absorbance at 595 nm of each well. The actual calcium concentration was equal to the corresponding calcium concentration of the sample obtained from the standard curve multiplied by the dilution factor. The fold change of calcium concentration was calculated and normalized to the control group.

### Lactate Dehydrogenase (LDH) Release

The release of lactate dehydrogenase into the extracellular space/supernatant is regarded as an important feature of the break of cell membrane integrity (Kumar et al., 2018; Parhamifar et al., 2019). For *in vitr*o analysis, the LDH cytotoxicity assay kit (Beyotime, Shanghai, China) was used to measure LDH released from necrotic cells in different groups. In brief, cell cultures were centrifuged at 400 *g* for 5 min and then cell-free culture supernatants were harvested from each well of the plate and incubated with the working reagent mixture for 30 min at RT according to the manufacturer’s instructions. For the *in vivo* analysis, the LDH cytotoxicity assay kit (Jiancheng Institutes, Nanjing, China) was used according to the manufacturer’s instructions. In brief, the eyes were quickly removed from anesthetized rats and then the rats were sacrificed by decapitation. The retinae were homogenized in 0.86% NaCl by sonication before incubation with the working reagent mixture for 30 min at 37°C. The optical density of each well in the assay, which in proportional to both the LDH activity and percentage of necrotic cells, was measured at a wavelength of 490 or 450 nm with a microplate reader. The percentage of necrotic cell death was calculated from four independent experiments by measuring the optical density of the treated group minus control group/LDH releasing, reagent treated group minus control group.

### Propidium Iodide (PI) Staining

Propidium iodide staining was used to identify cells undergoing necrosis (Jiang et al., 2014; Ding et al., 2015; Shang et al., 2017). Cell cultures on the coverslips were washed twice with PBS at the indicated time points and incubated with 10 μg/mL PI-dye solution in 5% CO_2_ incubator at 37°C for 15 min. For animal experiments, rats were euthanized 30 min prior to the indicated time-point and 5 μL 10 μg/mL PI-dye was intravitreally injected. The eye balls were separated before animal sacrifice. The eyes were frozen in liquid nitrogen vapor and cryosectioned followed by washing three times in PBS buffer and covered with an anti-fading mounting medium containing DAPI (Vector Laboratories, Burlingame, CA, United States). Images acquired with a fluorescence microscope using the same exposure time were captured for five random fields of each group. The percentages of PI-positive cells are equal to the number of PI-positive cells divided by the number of DAPI-positive cells, which was analyzed in every intact captured image using Image J software.

### Immunofluorescence Staining

At the indicated time points, cell cultures on the coverslips were washed three times with PBS and fixed with 4% PF for 20 min. After being washed in PBS three times again, the coverslips were blocked at RT in blocking buffer, i.e., PBS containing 5% normal bovine serum and 0.3% Triton X-100 for 1 h. The coverslips were then incubated with combinations of the primary antibodies against the following targets, overnight at 4°C: Pin1 (1:100, 3722S, Cell Signaling, Danvers, MA, United States), p-CaMKII (1:100, bs1647R, Bioss, Beijing, China), Map2 (1:200, M4403, Sigma, St. Louis, MO, United States). The coverslips were moved to RT for 30 min on the following day, washed three times, and incubated with Alexa-conjugated secondary antibodies (1:200, Jackson ImmunoResearch, West Grove, PA, United States) at RT for 2 h with fluctuation. The coverslips were washed three times and then covered with Vectashield mounting medium containing DAPI. All the staining procedures were in parallel and images were captured at five random fields of each coverslip using the same settings with a fluorescence microscope.

### Western Blot

Cell cultures were lysed on ice in RIPA buffer contained 1% protease inhibitors and 1% phosphorylated inhibitors (CWBIO, Beijing, China). The extracts were centrifuged at 12,000 × *g* for 20 min at 4°C to collect the supernatant and then a BCA assay was used here to determine the protein concentration. After unifying the protein samples, 5× loading buffer was added in the samples and the mixture was boiled for 5 min, centrifuged at 12,000 × *g* for 20 min at 4°C, and then the supernatant was harvest into a new tube. Twenty microgram proteins was loaded for each lane and separated using 4–15% or 10% SDS-PAGE gel and transferred onto a nitrocellulose membrane (GE Healthcare). The membranes were blocked in TBS-T containing 5% non-fat milk in for 1 or 2 h, at RT, and then incubated with the following primary antibodies overnight at 4°C: Pin1 (1:1,000, Cell Signaling), p-CaMKII (1:1,000, Bioss), CaMKII (1:1,000, 12666-2-AP, Proteintech, Rosemont, IL, United States), NR1 (1:500, bs-23343R, Bioss), GluR1 (1:500, bs-10042R, Bioss), GAPDH (1:5,000, AF0006, Beyotime, Beijing, China). After three washes in TBS-T, the membranes were incubated in TBS-T containing HRP-conjugated secondary antibody (1:5,000, Beyotime) at RT for 2 h, followed by three times wash in TBS-T buffer. And then the immunoreactive bands were visualized by low or high sensitivity chemiluminescence reagent (CWBIO). Integrated density values of specific proteins, which quantified by Image J software, were normalized to the GAPDH values.

### Simulation of Protein Binding Confirmation

The three-dimensional structures of proteins were built based on available protein crystal diffraction structure and frozen electron microscopic structures by homologous modeling (Swiss Model). To calculate the protein surface electrostatic potential energy, APBS (Figures were produced using PyMOL) (Baker et al., 2001) was used.

### Co-immunoprecipitation (Co-IP)

Primary cultured cells were lysed with a non-denaturing lysis buffer containing 1% protease and 1% phosphatase inhibitor and protein solution medium was centrifuged at 12,000 rpm (4°C) for 20 min. Four microgram CaMKII antibody (11533-1-AP, Proteintech) or IgG (B900610, Proteintech) was pre-incubated with protein A/G agarose beads (Beyotime) for 8 h at RT and washed with GLB+ buffer for five times. Then, 500 μg extracted protein was incubated with primary antibody coupled with protein A/G agarose beads at 4°C for 24 h with gentle fluctuating. After five times washed by GLB+ buffer, the protein-beads mixture coupled with 1× loading buffer was boiled for 5 min and centrifuged at 10,000 rpm for 5 min to get the supernatant. Finally, the bound proteins were analyzed by western blot.

### Real-Time Quantitative Polymerase Chain Reaction

After being washed three times in PBS, cell cultures were lysed in TRIzol^®^ (Invitrogen, Carlsbad, CA, United States) to harvest total RNA according to the manufacturer’s instructions. And then, 1 μg total RNA was used for cDNA synthesis using HiFiScript cDNA synthesis kit (CWBIO) according to the manufacturer’s instructions. Quantitative real-time polymerase chain reaction (RT-PCR) was performed using a sequence detection system (Prism 7500, Applied Biosystems Inc., Waltham, MA, United States). Amplification of specific PCR products was performed in triplicate in a total reaction volume of 10 μL-containing 2 μL cDNA template, forward and reverse primers (0.4 μM), and 2 × UltraSYBR mixture (Low ROX, CWBIO). The specific primers, purchased from Sangon Biotech Co., Ltd. (Shanghai, China) are listed in Table 1. GAPDH was used as an internal standard. Controls treated as above, without adding template, were included for each primer pair to check for any significant levels of contaminants. All threshold cycle (Ct) values were measured for a normalized fluorescence threshold of 0.02. All products produced similar amplification curves and single melt-curve peaks, indicating similar amplification efficiencies and a lack of non-specific amplification and primer-dimer formation. Relative mRNA levels were normalized to those of GAPDH and are calculated as 2^ΔΔ^*^*C*^*^t^.

**TABLE 1:** Primer sequence for real-time PCR.

**Gene/Protein**	**Forward primer (5′–3′)**	**Reverse primer (5′–3′)**
Grin1/NR1	ATGTGGTGGCTGTGATGCTGTAC	TTCCTCCTCCTCCTCACTGTTCAC
Grin2a/NR2A	GTGTGATGCCTGTCTGCGGATG	GCGTTGTTCTGTGACCAGTCCTG
Grin2d/NR2D	GCAAGGTCTTCGCCACCACTG	CAGCCGCTCCAGCATCTCAATC
Grin3a/NR3A	CAGCAGCAGCAGCAGTTCTTCC	AGGTTGAGAGGAGCCAGAGTTGTC
Gria1/GluR1	AGTCCAAGCCAGGTGTCTTCTCC	CTCTTCGCTGTGCCATTCGTAGG
Gria2/GluR2	CTCTTCGCTGTGCCATTCGTAGG	CAGTCCAGGATTACACGCCGTTC
Grik2/GluR6	GCTGCTATCTTCGGTCCTTCACAC	GTTGTCTGACACCTGGTGCTTCC
Grik4/KA1	ACGCCTTCCTGCTGGAGTCC	ACGCCTTCCTGCTGGAGTCC
Grm1/mGluR1	CCGCTCCAACACCTTCCTCAAC	ACCATGACACAGACTTGCCGTTAG
Grm3/mGluR3	CTATGTGTCCACCGTTGCCTCTG	GTCGTAGGACTTGCGGATGTTGG
Grm4/mGluR4	GTCACCTACACCAACCATGCCATC	CCTCAGCACCAAGCCACATTCG
GAPDH	GACATGCCGCCTGGAGAAAC	AGCCCAGGATGCCCTTTAGT

### Flash Electroretinogram (fERG)

Flash electroretinogram was used to monitor the visual function (Alarcon-Martinez et al., 2010). The RM6240 system (Chengdu Instrument Factory, Chengdu, China) was applied to fERG recording. After the drug treatment, the rats were dark-adapted for 12 h and then anesthetized under dim red illumination. Then, the recording electrode, reference and ground electrodes were inserted into the anterior chamber, the subcutaneous layer of the forehead and tail base, respectively. A bandpass filter of 10 Hz and 1.6 cd/s/m^2^ lash luminance was used. Each eye was exposed to flashes, three times, at 5-min intervals with the contralateral eye covered. All procedures were repeated at least four times. The amplitude of b wave was calculated from the bottom of a wave to the peak of b wave.

### Statistical Analysis

All experiments were repeated at least three times to ensure consistency of the results. Figure panels were assembled using Photoshop CC (Adobe Systems Incorporated, San Jose, CA, United States). The measurement data are expressed as the mean ± standard deviation (SD) and analyzed by one-way analysis of variance (ANOVA) which was carried out with GraphPad Prism 5 software (GraphPad Software Inc., San Diego, CA, United States). Statistical significance was set at *p* < 0.05.

## Results

### Glutamate Increased Calcium Concentrations in Retinal Neuron Cultures

**FIGURE 1:**
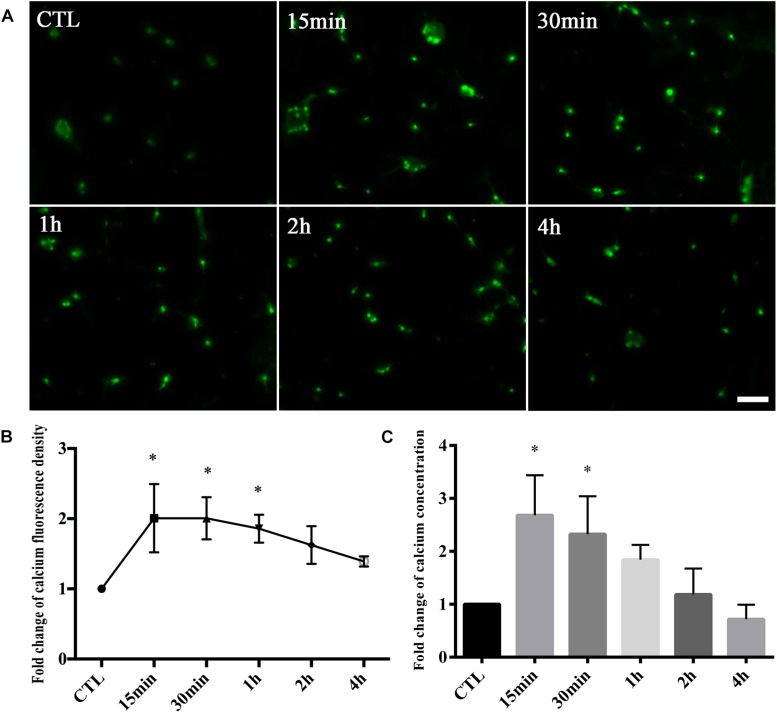
Calcium concentrations in retinal neuron cultures treated with 50 μM glutamate injury for different time points. **(A)** Calcium concentrations indicated by Fluo-4 signals. **(B)** Quantitative analysis of Fluo-4 fluorescence signal intensity. **(C)** The fold change of calcium concentrations was determined by cell calcium assay kit. Data were analyzed using one-way ANOVA. ^*^p < 0.05 vs. CTL group. These data are representative of results from five independent experiments. Scale bar = 20 μm in all panels.

We first tested whether changes in calcium occurred in retinal neurons following glutamate treatment. Fluo-4 was used as a calcium indicator. According to calcium imaging, it was clear that Fluo-4 signals were increased with increasing times of glutamate treatment compared to control groups (Figure 1A). Quantitatively, the average fold change of calcium fluorescence signal intensity was increased in 15-min glutamate treatment groups compared with control groups, and further declined after this time point (Figure 1B). Next, we used a cell calcium assay kit to directly test the fold changes of calcium concentrations in retinal neuron cultures. As shown in Figure 1C, the relative ratio of calcium concentration was increased in 15-min glutamate treatment groups, and then tended to gradually decrease. These results indicated that glutamate could increase calcium concentrations in retinal neurons.

### Glutamate Induced Changes in Calcium Concentration Rhrough iGluRs

**FIGURE 2:**
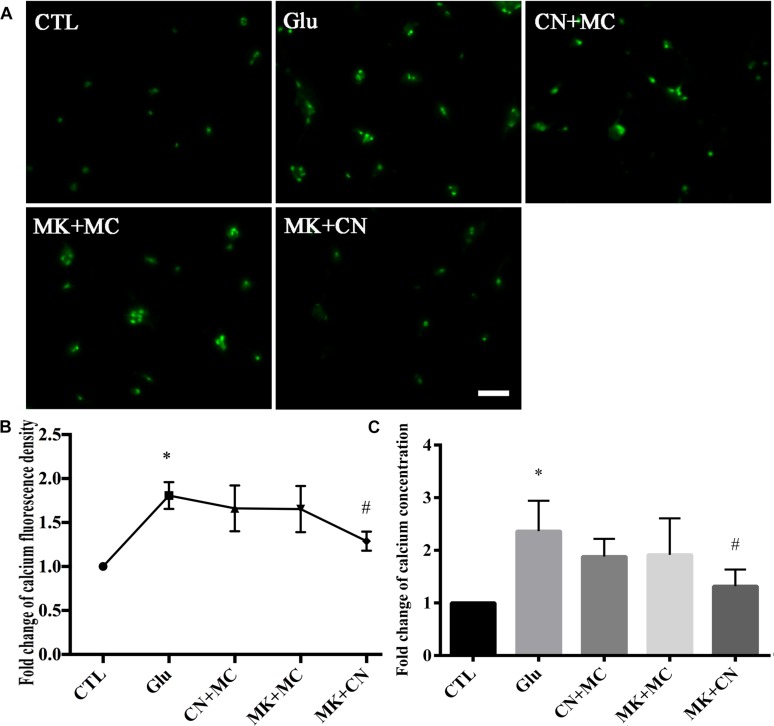
Calcium concentrations in retinal neuron cultures after different GluRs inhibition. **(A)** Calcium concentrations indicated by Fluo-4 signals. **(B)** Quantitative analysis of Fluo-4 signals intensity. **(C)** The fold change of calcium concentrations was determined by cell calcium assay commercial kit. MK, CN, and MC, which respectively block NMDAR, AMPA/KA receptor and mGluRs, were added to the cultures at a final concentration of 10, 10, and 50 μM, 30 min before 15 min glutamate insult. Experiments were repeated three times. Data were analyzed using one-way ANOVA. ^*^p < 0.05 vs. CTL group, ^#^ *p* < 0.05 vs. Glu group. Scale bar = 20 μm in all panels.

Since the average fold change of calcium fluorescence signal intensity was increased and peaked at 15 min after glutamate treatment, in this part of our study we investigated the battery of glutamatergic receptors responsible for glutamate-induced calcium changes in our retinal neuronal cultures at this time point. To identify whether NMDAR is involved in glutamate-induced changes in calcium concentration, before being treated with glutamate, retinal neurons were incubated with CN + MC for 30 min to block AMPAR/KAR and mGluRs. For inhibition of NMDAR and mGluRs, before treatment with glutamate, retinal neurons were incubated with MK + MC for 30 min. For inhibition of NMDAR and AMPAR/KAR, retinal neurons were pre-incubated with MK + CN for 30 min, and then treated with glutamate for 15 min. As shown in the Figure 2A, Fluo-4 signals intensity in MK+CN groups were much weaker compared with Glu, CN + MC, and MK + MC treatment groups. From quantitative results of calcium imaging (Figure 2B) and biochemical tests of the cell calcium assay kit (Figure 2C), it was also clear that the calcium concentrations in MK + CN groups were much lower compared with Glu, CN + MC, and MK + MC treatment groups. These results mean that it is possible that mGluRs activation cannot significantly change the calcium concentrations in our retinal neuron cultures after glutamate injury. It should be noted that although calcium concentrations in CN + MC and MK + MC treatment groups were somewhat decreased compared with Glu groups, this difference is not significant. Taken together, these results showed that glutamate may induce changes in calcium concentrations through iGluRs, which included both NMDAR and AMPAR/KAR.

**FIGURE 3:**
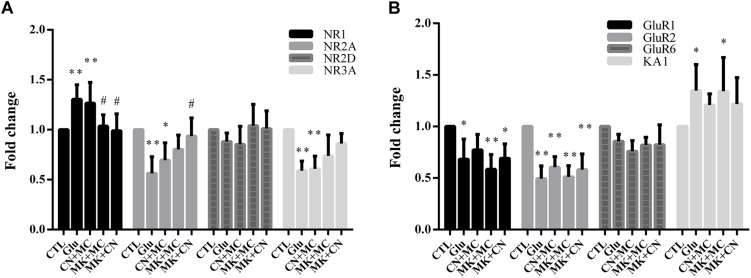
The mRNA level of various subtypes of ionotropic glutamate receptors after antagonist and glutamate treatment. **(A,B)** Fold change of various subtypes of glutamate receptors mRNA level detected by real-time PCR; MK, CN, and MC were used at a concentration of 10, 10, and 50 μM, 30 min before 15 min glutamate insult. To ensure consistency of the results, experiments were repeated four times. Data were analyzed using one-way ANOVA. ^*^p < 0.05, ^∗∗^*p* < 0.01 vs. respective CTL group, ^#^*p* < 0.05 vs. respective Glu group.

To investigate the expression of the various subtypes of glutamate receptors after antagonist and glutamate treatment, we first assessed the mRNA level of several NMDARs, AMPA/KA receptors. Then, we validated the protein expression levels of NR1, GluR1, which were widely reported to be involved in glutamate excitatory toxicity (Dijk and Kamphuis, 2004; Kaur et al., 2012; Chong et al., 2016). As shown in Figures 3A,B, real-time PCR results indicate that the levels of NR2A, NR3A, GluR1, and GluR2 mRNA were down-regulated after glutamate insult, while NR1 and KA1 mRNA levels were up-regulated. However, NR2D and GluR6 were not affected by glutamate exposure significantly. In addition, MK treatment seemed to attenuate the changes in NR1 and NR2A mRNA level. These results were similar to previous studies (Goebel and Poosch, 2001), i.e., that MK could affect the gene expression of NMDAR subunits. In addition, CNQX didn’t affect GluR1, GluR2, GluR6, and KA1 gene expression. Moreover, western blot analysis showed different results, that no change in NR1 and GluR1 protein level was observed within 15 min glutamate treatment (Supplementary Figure S1). The stable NR1 and GluR1 protein levels could be explained because our observations were conducted at early stages of necrosis, so the changes of protein levels can’t be tested at the early necrotic stages.

### Expression Changes of p-CaMKII, CaMKII, and Pin1 Following Glutamate Treatment

**FIGURE 4:**
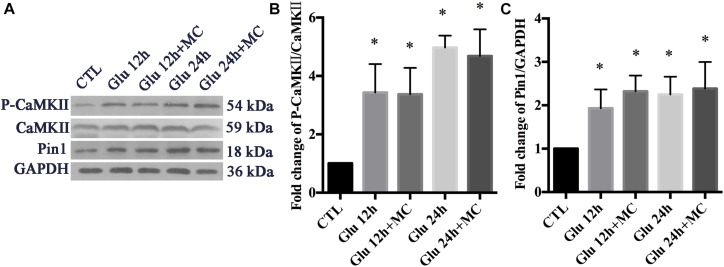
Expression of CaMKII, p-CaMKII and Pin1 in retinal neurons following glutamate treatment. **(A)** Western blot of CaMKII, p-CaMKII and Pin1 expression. **(B)** The statistical analysis of p-CaMKII/CaMKII expression. **(C)** The statistical analysis of Pin1 expression. MC was used at a concentration of 50 μM, 30 min before glutamate insult. *N* = 3 cultures. Data were analyzed using one-way ANOVA. ^*^p < 0.05 vs. CTL group.

We examined whether the expression of p-CaMKII, CaMKII, and Pin1 could be changed after glutamate treatment in retinal neurons. To exclude the underlying effect of mGluRs activities, MC groups were set as control groups. In our previously observed time points (15 min, 30 min, 1 h, 2 h), the expression of p-CaMKII, CaMKII, and Pin1 were not changed significantly following glutamate treatment (data not shown). We further extended our observed time points, because we had found expression changes of Pin1 in our previous studies (Wang et al., 2018a). Western blot results showed that the expression of p-CaMKII and Pin1 were significantly increased after 12 and 24 h of glutamate treatment (Figures 4A–C). These results indicated that glutamate could increase the expression of p-CaMKII and Pin1 in retinal neuron cultures.

### CaMKII Regulates Pin1 Activity in Retinal Neurons

**FIGURE 5:**
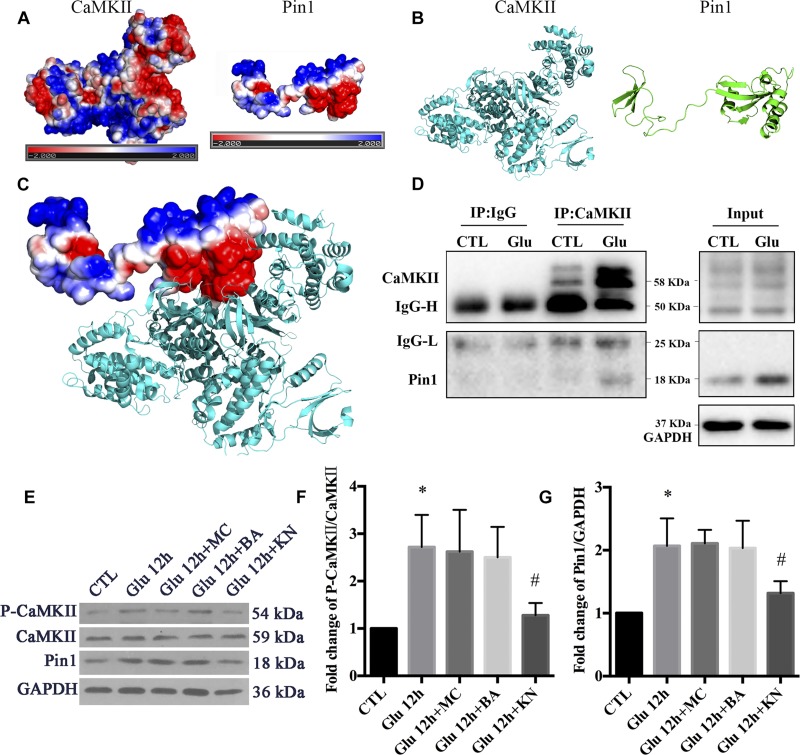
The regulatory role of CaMKII in Pin1 activity. **(A)** Three dimensional structure of proteins by homologous modeling (Swiss Model). **(B)** Protein surface electrostatic potential energy are calculated by APBS (Figures are produced by using PyMOL). **(C)** The initial binding mode is constructed by manually binding areas with opposite electrostatic potential of two proteins and then the protein complex is energy minimized using molecular dynamics software NAMD v2.12. **(D)** IP assay of CaMKII and Pin1. Pin1 was pull-down with CaMKII antibody suggested by a 18 KDa band as observed. **(E)** Western blot of CaMKII, p-CaMKII, and Pin1 expression after Glu, MC, BA, and KN treatment. **(F)** The statistical analysis of p-CaMKII/CaMKII expression. **(G)** The statistical analysis of Pin1 expression. MC, BA, and KN were used 30 min before glutamate treatment at a concentration of 50, 10, and 10 μM, respectively. *N* = 3 cultures. Data were analyzed using one-way ANOVA. ^*^p < 0.05 vs. CTL group, ^#^*p* < 0.05 vs. Glu group.

To examine the regulatory role of p-CaMKII in Pin1 activity, we first verified a previous report that CaMKII could bind with Pin1. The possible interaction between CaMKII and Pin1 was investigated using computer software. The electrostatic potential on CaMKII and Pin1 surface was calculated by APBS followed by energy minimization using NAMD v2.12. As shown in Figures 5A–B, both CaMKII and Pin1 displayed positive and negative electrostatic potential, which indicated that CaMKII could be bound to Pin1 (Figure 5C). We further performed Co-IP, and the results showed that CaMKII also interacted with Pin1 and the interaction was increased after glutamate treatment (Figure 5D). This result was correlated with previous reports that CaMKII could bind with Pin1 (Tatara et al., 2010).

To investigate the underlying effect of CaMKII in regulating Pin1 activity, we cultured retinal neurons with KN before glutamate treatment. We also added MC to the cultures as negative controls. In addition, BA was added to the cultures to investigate whether calcium was directly involved in the changes in Pin1 activity after glutamate treatment. Our results showed that KN efficiently blocked the CaMKII activity, which was shown by decreased p-CaMKII expression (Figures 5E,F). Furthermore, Pin1 activity was inhibited, as shown by decreased Pin1 expression after KN application (Figures 5E,G). However, the expression of p-CaMKII and Pin1 were not significantly changed in MC and BA groups compared with the glutamate groups (Figures 5E–G). These results suggest that the Pin1 activity might be affected by KN, but not MC and BA, in this glutamate model.

**FIGURE 6:**
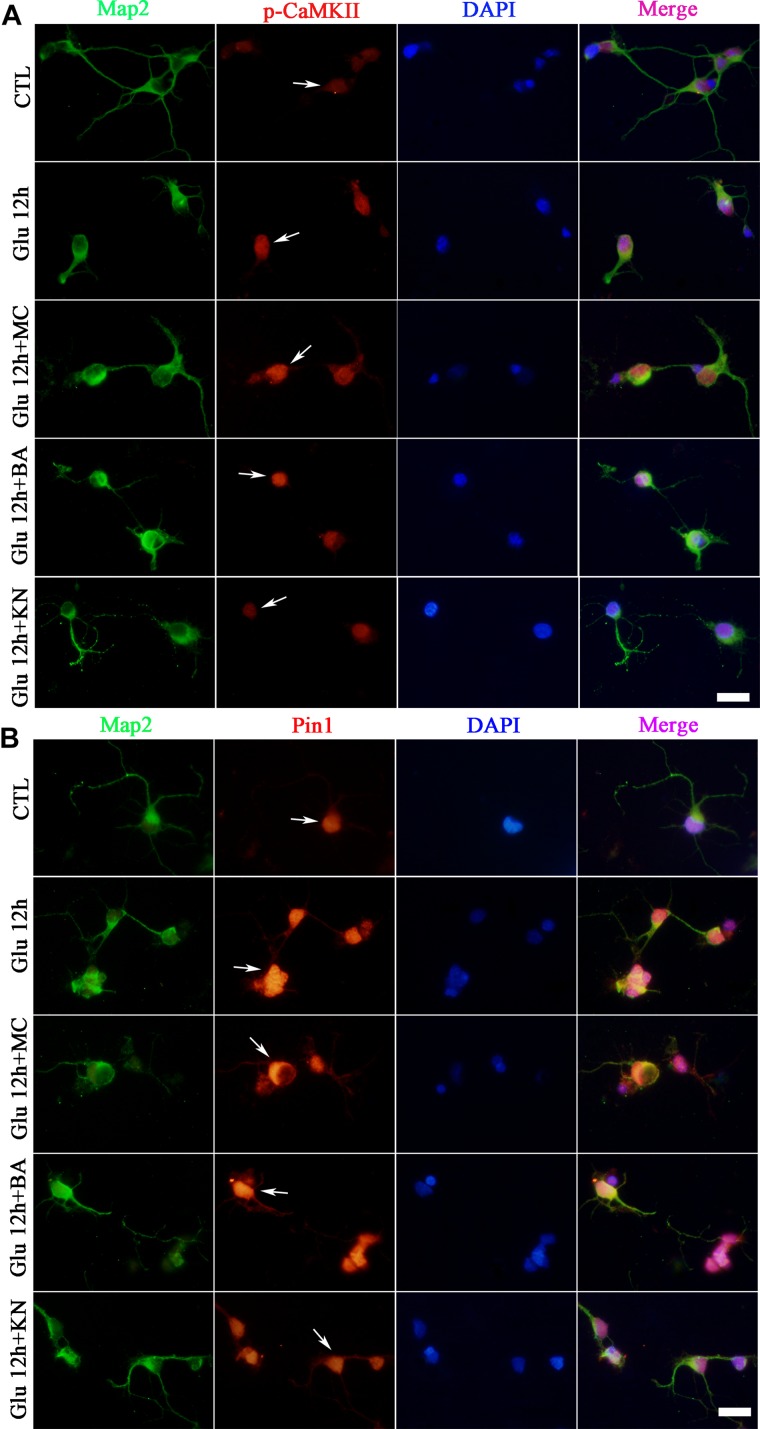
Immunofluorescence staining of p-CaMKII and Pin1 after glutamate and drugs treatment. **(A)** Immunofluorescence staining of p-CaMKII (red) and Map2 (green). **(B)** Immunofluorescence staining of Pin1 (red) and Map2 (green). *N* = 3 cultures. MC, BA, and KN were used 30 min before 12 h glutamate treatment at a concentration of 50, 10, and 10 μM, respectively. Scale bar = 10 μm in all panels.

Double immunofluorescence was further used to evaluate the effect of CaMKII in Pin1 activity. Immunostaining results showed that p-CaMKII was predominantly located in the cytoplasm and nucleus (Figure 6A). The immunofluorescence intensity of p-CaMKII showed a dramatic increase in the cytoplasm and nucleus after glutamate treatment (Figure 6A). However, the increased p-CaMKII declined after the KN application, compared with glutamate, MC and BA treatment groups (Figure 6A). Pin1 immunostaining was mainly present in the cytoplasm and nucleus. Pin1 expression was increased within the cytoplasm, nucleus, and slightly in dendrites (Figure 6B). The enhanced Pin1 level was decreased after the KN application, compared with glutamate, MC and BA treatment groups (Figure 6B). Altogether, these results confirmed that CaMKII activation may lead to the activation of Pin1 in retinal neurons after glutamate treatment.

### The Effect of CaMKII in RN

**FIGURE 7:**
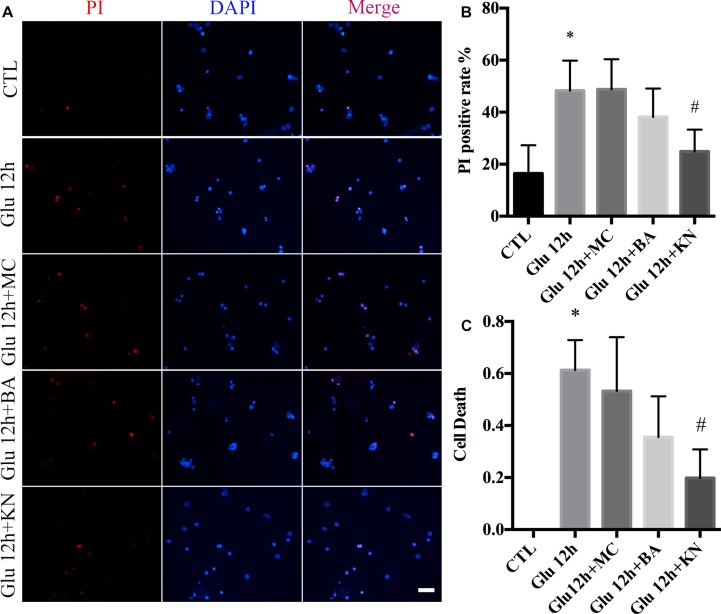
Glutamate-induced necrosis decreased by KN treatment. **(A)** Retinal necrotic neurons were stained with PI (red). Nuclei were counterstained with DAPI (blue). **(B)** Statistical analysis of PI-positive retinal neurons. **(C)** The percentage of necrotic neurons after glutamate treatment and pretreated with 50 μM MC, 10 μM BA, and 10 μM KN was determined by LDH release assay. *N* = 3 cultures. Data were analyzed using one-way ANOVA. ^*^p < 0.05 vs. CTL group, ^#^*p* < 0.05 vs. Glu group. Scale bar = 20 μm in all panels.

We evaluated the effect of KN-induced CaMKII inactivation in retinal neuronal RN. As shown in the PI staining and LDH assay, the necrosis in KN-treated retinal neurons was decreased, compared with glutamate and MC groups (Figures 7A–C). Although the necrosis in the BA groups was slightly decreased compared to glutamate, the difference was not significant (Figures 7A–C). These results indicated that inactivation of CaMKII by KN application can decrease the retinal neuronal RN induced by glutamate treatment.

### CaMKII Modulates Retinal Neuronal Necrosis in Retinal GCL and INL *in vivo*

**FIGURE 8:**
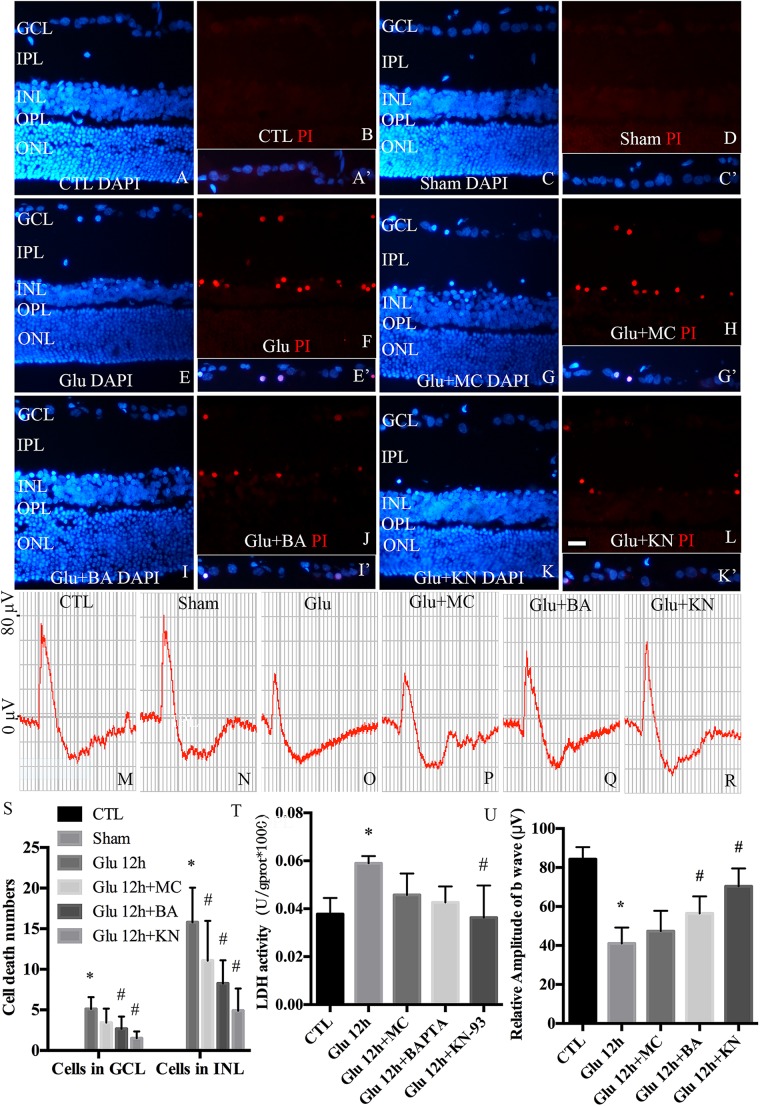
Necrosis and visual function in retina *in vivo.* **(A,C,E,G,I,K)** Nuclei were stained with DAPI (blue). **(B,D,F,H,J,L)** Retinal necrotic neurons were stained with PI (red). Nuclei and PI stained cells in GCL as indicated are shown in the merged frame areas (**A′,C′,E′,G′,I′,K′)**. **(M,N,O,P,Q,R)** Representative fERG results of groups treated with glutamate and pretreated with different drugs. **(S)** Statistical analysis of PI stained cells in GCL and INL in retina. **(T)** The percentage of necrosis cells were determined by LDH release assay. **(U)** Statistical analysis of the b wave amplitudes. ^*^p < 0.05 vs. CTL group, ^#^*p* < 0.05 vs. Glu group. Each group was composed of four animals. Data were analyzed using one-way ANOVA. GCL, ganglion cell layer; IPL, inner plexiform layer; INL, inner nuclear layer; OPL, outer plexiform layer; ONL, outer nuclear layer. Scale bar = 20 μm in all panels.

To examine the necrotic cells in rat retina *in vivo*, PI was intravitreally injected. Quantitative analysis of PI stained cells in the ganglion cell layer (GCL) and inner nuclear layer (INL) is shown in Figure 8S. Double immunofluorescence of DAPI and PI showed that the necrotic cells in GCL and INL were increased in glutamate groups (Figures 8E,F), compared with control (Figures 8A,B) and sham-operated groups (Figures 8C,D). PI staining results further demonstrated significant reductions in necrotic cells in both GCL and INL in KN (Figures 8K,L) and BA (Figures 8I,J) pretreatment groups. Necrotic cells in MC pretreatment groups (Figures 8G,H) were reduced in INL, but not GCL, compared with glutamate groups. LDH cytotoxicity assay *in vivo* was also conducted (Figure 8T). Compared with control and sham groups, increased LDH release was observed in the glutamate group. However, the increased LDH release was decreased in KN, but not MC and BA pretreatment groups. Taking these results together, they indicated that KN could provide a protective role in retinal cells in GCL and INL against glutamate induced neuronal necrosis.

fERG was performed to evaluate the visual function. fERG results showed that the amplitudes of b waves in glutamate groups (Figure 8O) were significantly decreased compared to control and sham groups (Figures 8M,N). Meanwhile, the amplitudes of b waves were enhanced in KN and BA treated groups (Figures 8Q,R). However, the amplitudes of b wave in MC groups were not significantly changed compared to the glutamate group (Figure 8P). Quantitative analysis of the b wave amplitudes is shown in Figure 8U. These results demonstrated that KN and BA have underlying protective effects on retinal function after glutamate treatment.

## Discussion

In the current study, we investigated the role of CaMKII in Pin1-modulated RN induced by glutamate in cultured rat retinal neurons and *in vivo*. In our previous study, we reported that Pin1-modulated RN occurred in cultured retinal neurons after glutamate injury (Wang et al., 2018a). In this study, we first demonstrated that glutamate might lead to calcium overloading mainly through iGluRs activation. Furthermore, CaMKII activation induced by overloaded calcium plays a regulatory role in Pin1 activity and subsequent RN. Finally, by using an animal *in vivo* model, we also demonstrated the important role of CaMKII in glutamate-induced RN in rat retina. These findings indicated the regulatory role of CaMKII in Pin1 activity and the involvement of the CaMKII-Pin1 pathway in glutamate-mediated excitotoxicity. These results will provide a potential target to protect neurons from necrosis in neurodegenerative diseases, such as glaucoma, diabetic retinopathy, and even CNS diseases.

In our previous study, we found that the Pin1-CAST/calpain2 pathway was involved in glutamate-induced retinal RN (Wang et al., 2018a). However, the mechanism of Pin1 activation induced by glutamate has been not fully elucidated. Neuronal responses to glutamate treatment are characterized by increased intracellular calcium concentration through GluRs activation (Mendez-Flores et al., 2016; Madji Hounoum et al., 2018). GluRs are classified into two main types: iGluRs and mGluRs. IGluRs (NMDAR and AMPAR/KAR) are ligand-gated ion channels that open upon the binding of glutamate, leading to the sustained influx of calcium (Wang and Qin, 2010; Madji Hounoum et al., 2016; Tiburcio-Felix et al., 2018). Activation of mGluRs, which are G-protein coupled receptors, leads to release of calcium from intracellular stores (Wang and Qin, 2010; Madji Hounoum et al., 2017). Some reports have indicated that all iGluRs are expressed in the retinal neuron cultures, while NMDARs are mainly responsible for glutamate induced retinal neurons death (Otori et al., 1998; Fang et al., 2010). Others have reported that non-NMDARs (AMPAR/KAR and mGluRs) also play key roles in glutamate mediated cell death in cultured retinal neurons (Dhingra and Vardi, 2012; Opere et al., 2018). To uncover the mechanism of Pin1 activation induced by glutamate, the distinct GluR subtypes involved in the glutamate-induced, first, retinal RN should be investigated. In this study, we used several antagonists to block GluR subtypes. Our aim is not to activate the NMDARs or AMPARs or mGluRs directly and investigate their roles in regulated necrosis (RN), but to inhibit two of them to investigate whether the un-inhibited glutamate receptors are involved in glutamate-induced RN under some neuronal diseases, such as glaucoma. So, we didn’t use the agonists to activate these receptors. While under a neuronal injury model, we used MK 801, CNQX, and MCPG, which are commonly used to block the activity of NMDARs, AMPAR/KA receptors and mGluRs, respectively. MK-801 was reported to be a highly potent antagonist of NMDA receptors. It could bind to the assembled helices of the transmembrane domain of NMDAR and block the channel ion conductance pore from the intracellular compartment (Berretta and Jones, 1996). Furthermore, MK-801 could significantly reduce glutamate-induced RGC death (Aihara et al., 2014). CNQX was first reported to be a competitive antagonist of kainate and quisqualate but not NMDA (Honore et al., 1988). Subsequent work revealed that CNQX had a high affinity for AMPARs primarily due to its slow rate of unbinding (Rosenmund et al., 1998). It has been reported that CNQX could reversibly block AMPA- and KA-evoked robust voltage responses in rabbit retinal slices (Zhou and Fain, 1995), decelerating the progression of RGCs dysfunction (Xiang et al., 2018). Subtypes of mGluRs share a common structural architecture with a large extracellular domain preceded by the seven membrane-spanning domains (Okamoto et al., 1994). mGluR1 and mGluR5 are coupled to the stimulation of the phosphatidylinositol hydrolysis/calcium signal transduction, whereas the others are linked to the inhibition of the CAMP cascade (Hayashi et al., 1994). MCPG has been reported to be a non-specific antagonist of group I (mGluR1 and mGluR5) and group II receptors (mGluR2 and mGluR3) (Kohlmeier et al., 2013; Yang et al., 2015), exhibiting little effect on either the NMDA receptor or the AMPA/kainate receptor (Jane et al., 1993). Results in Figure 2 directly demonstrate that iGluRs may be involved in the glutamate-induced rise of calcium. Furthermore, RT-PCR results also indicated that several iGluRs, such as NR1, NR2A, NR3A, GluR1, GluR2, and GluR6 mRNA level were changed after glutamate injury. NMDA receptor is a heteromeric ligand-gated ion channel composed of multiple receptor subunits (NR1, NR2, and NR3A). The NR1 subunit appears to be ubiquitous and necessary in order to have a functional and calcium-permeable receptor (Chen et al., 2004). Inclusion of NR3 subunits could cause a five- to ten-fold decrease in calcium permeability of traditional NR1/NR2 heteromers (Sasaki et al., 2002; Tong et al., 2008). Thus, increased NR1 and decreased NR3A levels may lead to the increased calcium concentration in retinal neuron cultures after glutamate injury. G1uR2 determines the calcium permeability of AMPA receptors (Chen et al., 2001; Harvey et al., 2001). AMPA receptors composed of G1uR1, G1uR3 or GluR4 (either alone or in combination) have permeability and bidirectional rectification effects on calcium, while the channel composed of G1uR2 and other subunits shows an opposite effect (Gouaux, 2004; Topolnik et al., 2005). Our results also showed a significant decrease of G1uR2 mRNA along with increased calcium concentration. In addition, although mRNA levels of NR1 and GluR1 were changed, the related protein expression levels remained unchanged, probably due to the short glutamate exposure time in our observation (Supplementary Figure S1). In contrast, mGluRs appear not to be significantly related to the regulation of calcium concentration in our model (Figure 2). However, we cannot exclude the possibility that part of the increased calcium could come from mGluRs, since inhibition of mGluRs partially reduced the fluorescence of the calcium indicator, although the difference is not significant. The reason may be that AMPA/KA and NMDA receptors are expressed in isolated retina cultures aged 3–8 days and are largely responsible for calcium flux across neuronal membranes (Otori et al., 1998; Madji Hounoum et al., 2016). Another plausible reason may be that the expression of mGluRs in the neonatal retina is low, but greater in adults (Ghosh et al., 1997). Since we used the neuronal cultures at 7 days, it is possible that the relative stability of calcium concentration in the MK + CN group compared with the control group is partly due to the low expression of mGluRs.

Glutamate is a major physiological neurotransmitter in the central neuron system, its appropriate level at the synapses is strictly controlled by glutamate uptake and cycling mechanisms (Danbolt, 2001). Glutamate transporters in glial cells play a major role in keeping extracellular glutamate concentration below neurotoxic levels (Madji Hounoum et al., 2017). Glutamate transporters could remove glutamate from the extracellular space by taking glutamate up into neurons and glia cells, it also reported to be involved in reverse uptake, releasing glutamate into the extracellular space (Grewer et al., 2008). Under pathological conditions, the impaired function of glutamate transporters leads to the existence of a high concentration of glutamate in the synaptic cleft, ultimately resulting in neuronal death (Lewerenz and Maher, 2015). In this study, we cannot exclude the possibility that part of the glutamate effect is in fact related to the activity of the glutamate transporters since antagonist treatment could not block all the changes in glutamate-induced calcium concentration. However, in this study, we used retinal neuron cultures in which few glial cells exist. Thus, it’s understandable to presume that the functional roles of glial glutamate transporters in our neuronal cultures can be neglected.

CaMKII is a kinase that can be regulated by CaM, a calcium-binding protein (Tatara et al., 2010). The interaction of Pin1 and CaMKII has been reported (Tatara et al., 2010). However, the regulatory role of CaMKII in Pin1 has not been investigated. We would like to know whether intracellular calcium directly participates in glutamate-induced CaMKII and Pin1 expression. As observed, the *in vitro* results showed that chelation of intracellular calcium with BAPTA-AM had no statistically significant effect on CaMKII and Pin1 expression after glutamate treatment. In addition, although the RN in the BAPTA-AM group was slightly reduced, it was also not significant. The plausible explanations for this phenomenon may be that: (1) loading cultured neurons with BABTA-AM might cause ER-specific stress, which would trigger abnormal proteins accumulation (Hofer and Machen, 1993; Pozzo-Miller et al., 1997); and (2) BAPTA-AM is a fast process with a half-time for maximal loading of a few minutes (Tymianski et al., 1997; Paschen et al., 2003). In our study, the change of calcium concentration lasted up to 1 h. Therefore, it could be assumed that the effect of calcium on neurons exceeds the effective function of BAPTA-AM. However, *in vivo* studies showed that BAPTA-AM treatment was able to reduce RN in retinal GCL and INL, and recover the visual function impaired by glutamate. The reason of different results between *in vitro* and *in vivo* studies may be that loading primary neuronal cultures with BAPTA-AM can induce neurotoxic effects as previously reported (Paschen et al., 2003). Another reason may be that cultured neurons appear to be more sensitive to excitotoxic injury than other neurons *in vivo* (Ullian et al., 2004; Mitra et al., 2013). Therefore, BAPTA-AM application tends to be sufficient to rescue the neuronal survival and visual function *in vivo*.

**FIGURE 9:**
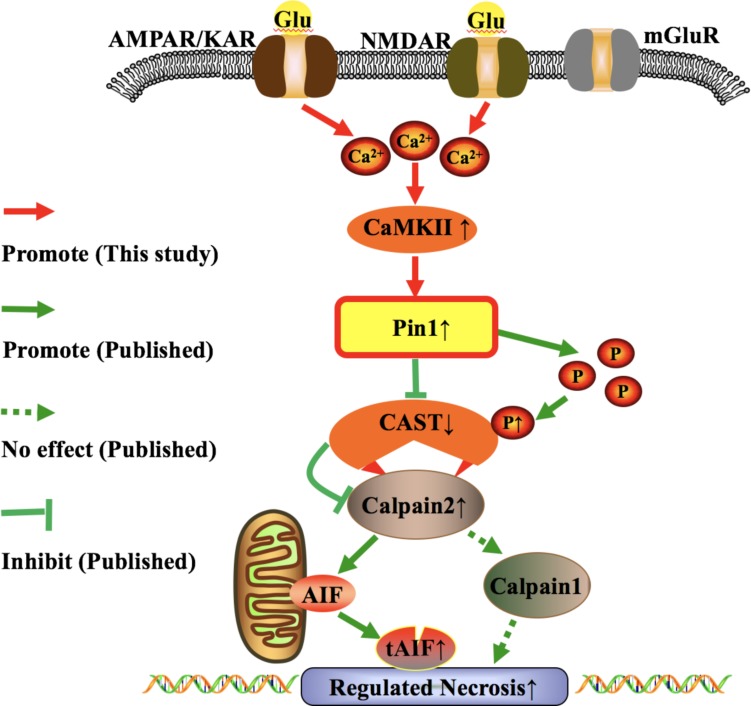
Possible molecular pathway underlying the effect of glutamate in RN of retinal neurons. The role of iGluRs-CaMKII-Pin1-CAST/calpain pathway induced by excessive glutamate in RN of retinal neurons.

It has been reported that KN-93 could prevent the activation of CaMKII (Takeuchi and Yamamoto, 2015; Li J. et al., 2016). In our next study, we found that inhibition of CaMKII by KN-93 could reduce the activity of Pin1 and retinal neuronal RN. In fact, our results were consistent with other reports that inhibition of CaMKII reduced the activity of calpain (Menconi et al., 2004; Kong et al., 2017). Pin1 has been considered as a positive up-regulator of calpain in our previous research (Cheng et al., 2018; Wang et al., 2018a). Taking these reports together, it is reasonable to conclude that CaMKII-Pin1-calpain activation is a key regulatory molecular pathway responsible for retinal RN induced by glutamate. In this pathway, Pin1 serves as a connecting link between CaMKII and calpain (Figure 9). In addition, peptidyl-prolyl *cis*/*trans* isomerase parvulin 17 (Par17) is another subtype of the PPIases family. It has been reported that Par17 is able to interact with CaM at the 25-residue elongation of the Par17 N-terminus (Burgardt et al., 2015). Whether Pin1 could directly interact with CaM, the up-regulator of CaMKII, also needs to be further investigated. Also, the sites of Pin1 that interact with CaMKII and/or CaM need to be clarified by further research. Overall, by using primary retinal neuronal cultures and the rat model, we obtained novel evidence supporting the CaMKII mediated Pin1 activation and RN under conditions of excessive glutamate.

In conclusion, our results indicated that CaMKII is an up-regulator of Pin1 and responsible for the RN induced by glutamate. These results provide further understanding of the regulatory pathway of RN and a complementary mechanism for calpain activation mediated RN.

## Ethics Statement

All experimental procedures used in the present study were approved by the Ethics Committee of the 3rd Xiangya Hospital of Central South University in accordance with the U.S. National Institutes of Health (NIH) Guidelines for the Care and Use of Laboratory Animals.

## Author Contributions

KX and JH designed the study. SW and LL conducted the experiments, analyzed the data, and prepared the manuscript and images. YH, MW, BJ, DC, HZ, DJ, and FL conducted the experiments, prepared the manuscript and images, collected and analyzed the data and literature. KX, SW, and LL revised the manuscript. All authors read and approved the final version of the manuscript. All authors agreed to be accountable for all aspects of the study in ensuring that questions related to the accuracy or integrity of any part of the work are appropriately investigated and resolved.

## Conflict of Interest Statement

The authors declare that the research was conducted in the absence of any commercial or financial relationships that could be construed as a potential conflict of interest.

## Supplementary Material

FIGURE S1 Expression of NR1 and GluR1 after antagonist and glutamate treatment. **(A)** Western blot assay of NR1 and GluR1 protein expression in glutamate group and iGluRs inhibition group. **(B)** The statistical analysis of NR1 and GluR1 expression. MK, CN and MC were used at a concentration of 10, 10, and 50 μM 30 min before 15 min glutamate insult. To ensure consistency of the results, experiments were repeated three times. Data were analyzed using one-way ANOVA.
