# Supplementary figures and images for: Pin1 Is Regulated by CaMKII Activation in Glutamate-Induced Retinal Neuronal Regulated Necrosis
Source: Front Cell Neurosci. 2019 Jun 25;13:276. doi: 10.3389/fncel.2019.00276 (PMC6603237; doi:10.3389/fncel.2019.00276)

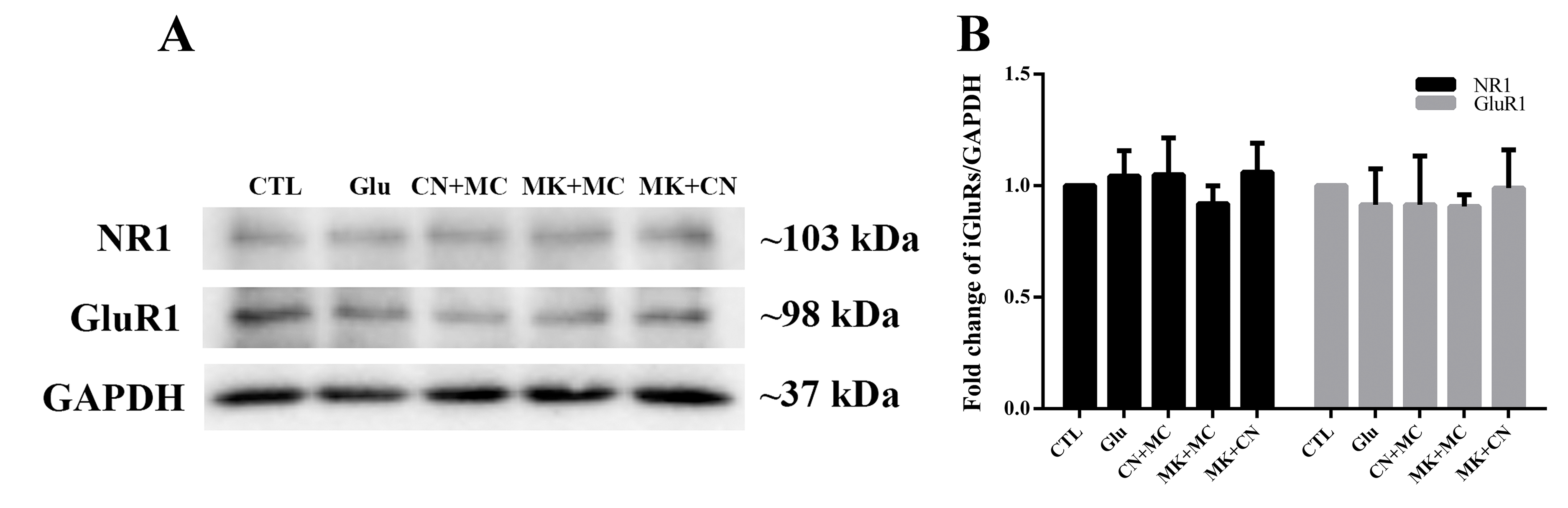

Supplement: FIGURE S1 — Expression of NR1 and GluR1 after antagonist and glutamate treatment. (A) Western blot assay of NR1 and GluR1 protein expression in glutamate group and iGluRs inhibition group. (B) The statistical analysis of NR1 and GluR1 expression. MK, CN and MC were used at a concentration of 10, 10, and 50 μM 30 min before 15 min glutamate insult. To ensure consistency of the results, experiments were repeated three times. Data were analyzed using one-way ANOVA. [file Image_1.TIF]
